# Predicting Short Time-to-Crime Guns: a Machine Learning Analysis of California Transaction Records (2010–2021)

**DOI:** 10.1007/s11524-024-00909-0

**Published:** 2024-09-05

**Authors:** Hannah S. Laqueur, Colette Smirniotis, Christopher McCort

**Affiliations:** 1 Violence Prevention Research Program, Department of Emergency Medicine, University of California, Davis, USA; 2 California Firearm Violence Research Center, Davis, USA

**Keywords:** Firearm transactions, Crime guns, Short time-to-crime, Random forest, Risk prediction, Variable importance

## Abstract

Gun-related crime continues to be an urgent public health and safety problem in cities across the US. A key question is: how are firearms diverted from the legal retail market into the hands of gun offenders? With close to 8 million legal firearm transaction records in California (2010–2020) linked to over 380,000 records of recovered crime guns (2010–2021), we employ supervised machine learning to predict which firearms are used in crimes shortly after purchase. Specifically, using random forest (RF) with stratified under-sampling, we predict any crime gun recovery within a year (0.2% of transactions) and violent crime gun recovery within a year (0.03% of transactions). We also identify the purchaser, firearm, and dealer characteristics most predictive of this short time-to-crime gun recovery using SHapley Additive exPlanations and mean decrease in accuracy variable importance measures. Overall, our models show good discrimination, and we are able to identify firearms at extreme risk for diversion into criminal hands. The test set AUC is 0.85 for both models. For the model predicting any recovery, a default threshold of 0.50 results in a sensitivity of 0.63 and a specificity of 0.88. Among transactions identified as extremely risky, e.g., transactions with a score of 0.98 and above, 74% (35/47 in the test data) are recovered within a year. The most important predictive features include purchaser age and caliber size. This study suggests the potential utility of transaction records combined with machine learning to identify firearms at the highest risk for diversion and criminal use soon after purchase.

**Supplementary Information:**

The online version contains supplementary material available at 10.1007/s11524-024-00909-0.

## Introduction

Gun-related crime continues to be an urgent public health and safety problem in the United States. The firearm homicide rate increased by close to 35% in 2020 from the year prior [1] and rose another 8% in 2021, reaching a 29-year high [2]. In that year, firearms were used in 81% of the more than 20,000 homicides, the highest proportion reported in over 50 years [1]. This rise in homicides coincided with record-high firearm sales, which researchers have linked to increased gun violence [3, 4]. It also coincided with a significant increase in firearms recovered in crimes shortly after legal purchase [5]. The rapid diversion of a firearm from sale to criminal use, i.e., a short “time-to-crime,” is a frequently used indicator of likely illegal activity by dealers, purchasers, or traffickers [6, 7].

An understanding of the relative risks for diversion and criminal use among firearm sales can inform efforts to reduce the flow of guns into illicit markets and criminal hands [8]. However, much of the research examining how firearms move from the primary market to illegal possession and criminal use is dated and limited [7, 9]. Congressional restrictions on the Bureau of Alcohol, Tobacco, Firearms and Explosives (ATF) from record sharing have precluded gun trace data research, other than at the local jurisdiction level, since the early 2000s. Additionally, identifying risk factors associated with the firearms that end up being used in crime, as compared to the majority that do not, is only possible in the handful of states that record and maintain firearm purchase data [8]. Research in California in the early 2000s combined ATF trace data on firearms recovered in crimes by law enforcement and the state’s archives of individual transactions to examine the associations of purchaser, firearm, retailer, and community characteristics with firearms used in crime [10, 11]. Similar work was done in Maryland [12], another state that maintains handgun transaction records. In both contexts, these studies found a number of consistent crime gun risk factors including firearms that are semiautomatic, medium to large caliber, and inexpensive; purchasers that are non-white, young, and female; and retailers that are licensed as pawnbrokers and that have a disproportionate number of purchase denials following a background check relative to their total sales [8, 10, 11]. In work related to the present study, we conducted a survival analysis [13] using updated crime gun data for the state of California [14], linked to firearm transaction records, and confirmed many of the previously documented associations. We also examined variables not previously studied, and found, for example, that firearms reported stolen were nine times more likely to be recovered in crime.

The present study is the first study to employ a machine learning approach to identify which transactions are at high risk for recovery shortly after purchase, and the most important purchaser, firearm, and retailer predictors of this risk. Specifically, we rely on datasets that include close to 8 million firearm transaction records in the state and approximately 380,000 records of recovered crime guns from 2010 to 2021 to predict whether a firearm was recovered within a year of purchase (0.2% of transactions) and whether the firearm was recovered within a year of a violent crime (0.03% of transactions).

Overall, our models show good discrimination between the small fraction of guns recovered shortly after purchase and the vast majority that are not, and we are able to relatively accurately identify firearms at extreme risk for diversion from the legal market into criminal hands. Though these risk prediction models are largely “proof-of-concept,” we suggest risk prediction such as this could potentially aid violence prevention, for example, by supporting current efforts to prevent straw purchasing or supplementing the background check process.

## Methods

### Data

The principal data for this study are California Dealer Records of Sale (DROS) firearm transaction records from 2010 to 2020 (*n* = 7,818,362) and gun trace records for 380,619 recovered crime guns from 2010 to 2021. Both sets of data are maintained in the California Department of Justice (CA DOJ) Automated Firearm System (AFS). In California, all sales and transfers of firearms must be done through a federally licensed firearms retailer (FFL). These include transfers between private parties, gun show sales, gifts, loans, and redemption of pawned or consigned weapons. Retailers are required to electronically transfer all details of the transaction, including information on the firearm, transferee, and retailer, to CA DOJ, where the information is stored. The AFS database contains DROS records for all handgun transactions since 1996 and transactions for rifles and shotguns since 2014.

In 2002, California enacted the nation’s first statewide crime gun tracing bill, which mandates that all firearms used in a crime, suspected to have been used in a crime, illegally possessed, or found by law enforcement, must be submitted to the CA DOJ for the purpose of tracing through ATF (Calif. Penal Code §11108[a]). CA DOJ is required to maintain the records for at least 10 years. It is from these data that we record crime gun recovery.

To avoid bias due to missing crime gun recovery data, our analyses focus on firearm transaction since 2010, though we use the full set of transaction records dating back to 1996 (*n*=10,662,943) to generate features related to individuals’ purchase histories. Our primary models include handguns and long guns. However, given long gun data are only consistently available beginning in 2014, and over 70% of crime gun recoveries are handguns, we conduct secondary analyses restricting the dataset to handgun transactions only.

#### Outcomes

Our primary outcomes are crime gun recovery within 1 year of the transaction and violent crime gun recovery within a year. We were interested in estimating this short “time-to-crime,” as this is a commonly used indicator of potential illegal activity by dealers or traffickers, with less than 3 years between the first retail sale and recovery in a crime generally considered an indicator of possible illegal activity and a time of less than 1 year a very strong indicator [6, 7, 15].

Among transactions from 2010 to 2020, a total of 15,945 firearms (0.2% of transactions) were recovered within one year of purchase (2010-2021). A total of 2,132 (0.03%) were recovered in association with a violent crime. Violent crimes were categorized based on the CA DOJ crime categories and include assault (45.5%), homicide (26.0%), robbery (15.6%), threats (9.8%), kidnapping (1.8%), and sexual violence (1.2%).

#### Predictor Variables

We generated and included a total of 81 purchaser, firearm, transaction, retailer, and community-related predictor variables. Purchaser-level features, derived from DROS, included purchaser sex, race/ethnicity, and age at the time of the transaction. In a secondary analysis, we excluded race/ethnicity from the model. The CA DOJ provided us with criminal history records, maintained in their Automated Criminal History System (ACHS), for all individuals with a record of transaction in DROS. These criminal history data include all arrests and convictions within the state since 1981. We included the number of prior violent, property, firearm-related, alcohol-related, and drug-related arrests associated with the purchaser at the time of the transaction.

We included several features from DROS related to the firearm, such as make, model, and caliber. We categorized caliber size into small (e.g., .22, .25, .32), medium (e.g., .38, .3, 9 mm), and large (e.g., .40, .44, .45) for handguns. Long guns were classified as rim-fire rifles, center-fire rifles, frame/receiver only rifles, and shotgun (410, not 410, and frame/receiver). We included a feature specifying the firearm category (semiautomatic pistol vs revolver vs unknown) and an indicator for “inexpensive” firearm, which we proxied by the manufacturer, selecting the bottom quantile of median prices found in the *Blue Book of Gun Values* [16]. Prior crime gun research has documented a positive association between larger caliber handguns and “cheap” handguns and crime gun recovery [8, 10–13, 17].

We generated and included several features related to the transaction and purchaser’s prior transaction history. The primary transaction characteristic thought to be related to gun trafficking (and crime gun recovery) is multiple sale transactions—the purchase of multiple guns by one individual within a short period of time, usually defined as 30 days [12]. However, California limits buyers to one firearm purchase every month. We nonetheless hypothesized that frequent purchasing within the last few months might be an indicator of problematic activity and thus included a variable for the number of transactions a purchaser made in the 6 months prior to a given transaction. We also included the number of prior firearm purchases in the last year, 5, 10, and 20 years. We included previous attempted purchases that were denied. Most often denials are issued because the would-be purchaser has a prohibiting criminal history. We hypothesized that risk would likely be highest for denied purchases in close proximity. We included denials within 90 days, 180 days, 1 year, and 5 years. Finally, we included binary indicators for whether the firearm was purchased at a gun show, whether the transaction was a sale, an administrative denial of sale, a voluntary registration, pawn redemption, or law enforcement acquisition.

We included several predictor variables related to the retailer. We included features summarizing the dealer’s prior sales in the past calendar year: the proportion of sales in the past year that were pawn, the proportion that were administrative denials, and the proportion of prior sales that resulted in crime gun recovery. We geocoded both purchaser and dealer premise address and included the distance traveled in kilometers from the purchaser’s home address to dealer’s premise address.

Finally, we included a number of community variables associated with both the dealers’ address and purchasers’ address. These community features included the US Census and American Community Survey Social Vulnerability Index (SVI) sub-scales on the relative vulnerability of a census tract. We included the overall SVI and the SVI in relation to socioeconomic status, racial and ethnic minority status, household characteristics, and housing type and transportation. Firearm violence has been shown to concentrate in urban neighborhoods with high social vulnerability, as measured by SVI [18]. Further, in our multivariate survival analysis of crime gun recovery, we found that a purchase made by an individual living in a census tract with higher SVI for socioeconomic status was positively associated with crime gun recovery [13]. Additional community characteristics in the model included Rural–Urban Commuting Area (RUCA) codes for the associated county (most urban vs not and most rural vs not) and city crime rates reported in the FBI Uniform Crime Reports. We relied on the Law Enforcement Agency Identifiers Crosswalk (LEAIC), which links Originating Agency Identifier (ORI) crime reports to Federal Information Processing Standards (FIPS) place codes. We generated a time-varying past year crime rate associated with both the purchaser and dealer premise address. We implemented a 1-km buffer radius for geocoded addresses that fell outside a FIPS place. Approximately 10% of addresses did not have associated FIPS places and therefore had missing crime data.

**Table 1:** Predicting any crime gun recovery within 1 year

Threshold	Sensitivity	Specificity	TPR	FPR	FNR	Youden	*F*-score
0.42	0.740	0.800	0.740	0.200	0.260	0.540	0.015
0.50	0.631	0.879	0.631	0.121	0.369	0.510	0.021
0.62	0.447	0.951	0.447	0.049	0.553	0.397	0.035
0.85	0.101	0.997	0.101	0.003	0.899	0.098	0.076

We conducted a sensitivity analysis that simplified and removed several features such that there were a total of 50 predictors (vs 81 in the primary models). Though machine learning algorithms and random forest (FR), in particular, are robust to the inclusion of a large number of predictor variables, including correlated predictors [19], variable correlation can impact variable importance measures [20]. In the reduced-variable model, we used only the composite Social Vulnerability Index (SVI) measure, dropping its component pieces. We also consolidated features that were engineered to capture events over different time frames. We instead included a feature indicating any prior arrest for each crime type within 30 years and any prior purchase denial (rather than arrests and denials over different time spans). We included just two features capturing prior purchases: the number in the past 6 months and the number in the past 10 years.

A summary table of all predictors and their average values for firearms recovered within a year of transaction and those not recovered within a year are provided in the supplement (Table A1).

### Prediction Algorithm

We implemented a random forest (RF) classification model [21] to predict crime gun recovery. RF is among the most popular and strongest performing classifiers [22] and has been shown to perform well on imbalanced data (i.e., data with a rare outcome) [23]. We used this approach to predict firearm suicide within 1 year of sale [24], and it also has been successfully applied in a number of criminal justice contexts such as predicting risk of re-arrest among parolees [25].

RF consists of a large number of individual decision trees, each of which is built from a random sample (sampled with replacement) from the training data (i.e., data used to build the model, but not used in model evaluation). Each tree creates binary splits in the data, based on a sample of predictor variables, drawn randomly at each partition, and selects the purest split—i.e., the split that results in the most class separability. Each tree is grown, without pruning, until either purity (i.e., homogeneity) or node size 1 is reached. Each tree then predicts the outcome value for the remaining observations in the training set. Finally, the classification trees are aggregated to create the RF algorithm, and each observation receives a predicted probability based on the proportion of trees that assign it to the positive class (crime gun). The probability or score can then be converted to a single outcome class (0/1) based on a “decision threshold.” The default threshold is majority rule (i.e., an observation with $> 50\%$ of the tree “votes” is classified as a 1) [26].

The two primary tuning parameters for RF are the number of predictor variables randomly selected at each binary split (*mtry*), and the number of trees in the forest (*ntree*). We selected the optimal *mtry* and *ntree* by maximizing the area under the receiver operating characteristic curve (AUC). We implemented the RF using the caret package in R [27], which by default employs bootstrap resampling for hyper-parameter tuning. We allocated a random sample of $70\%$ of the data as the training set and used the remaining $30\%$ as test set data. This test set data was strictly unseen throughout the entire model training and hyper-parameter tuning processes.

Given the rarity of our outcomes, we incorporated random under-sampling, a common approach for prediction in the context of imbalanced data [28, 29]. Random under-sampling balances the training data by randomly discarding instances of the majority class. This helps to avoid the problem of the algorithm ignoring the minority class and improves its ability to identify and isolate the signal of interest [30].

We implemented stratified under-sampling within the RF algorithm. Thus, for each tree in the forest, a bootstrapped sample of the same size was taken from each class (stratum) to create a balanced dataset with which to grow each tree. Importantly, though we constructed the models using training data with balanced classes, the data used to *test* the algorithm’s performance remained unbalanced.

**Fig. 1:**
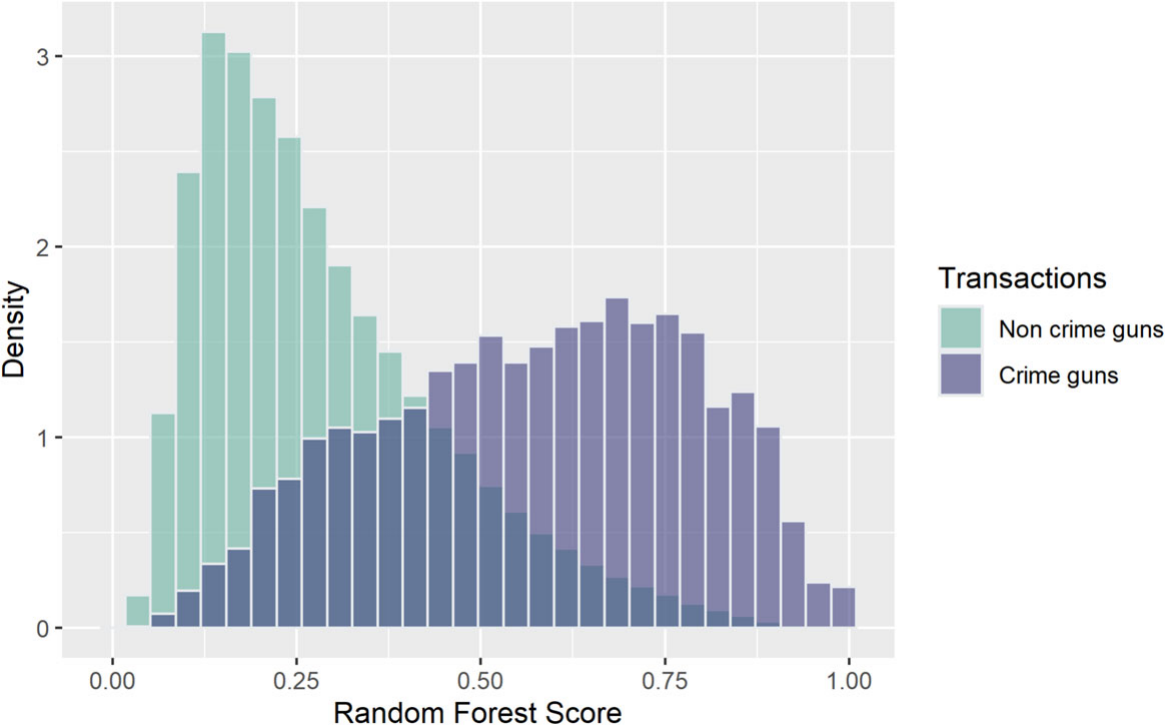
Density plot: RF predictions by outcome

### Algorithm Evaluation

We evaluated the model in several ways. We report test set AUC, which describes the algorithms ability to distinguish between positive and negative classes. A classifier that can perfectly distinguish between positive and negative cases would have an AUC of 1; an AUC score of 0.5 suggests that the model performs no better than random chance. We also present sensitivity (the true positive rate), specificity (the true negative rate), and metrics that combine sensitivity and specificity that are commonly used for imbalanced classification problems [31], including *F* measure ((2 $\times$ sensitivity $\times$ specificity)/(sensitivity $+$ specificity)) and Youden’s index (sensitivity $+$ specificity $- 1$). We report these metrics for a range of thresholds including the default (.5), the threshold that maximizes *F*-score, and the threshold that maximizes Youden’s index. We also present the distribution of raw scores or predicted probabilities generated by the RF, examining the concentration of risk and the proportion of crime gun recoveries among transactions classified as highest risk.

**Table 2:** Predicting violent crime gun recovery within 1 year

Threshold	Sensitivity	Specificity	TPR	FPR	FNR	Louden	*F*-score
0.42	0.768	0.790	0.768	0.210	0.232	0.559	0.002
0.50	0.662	0.879	0.662	0.121	0.338	0.541	0.003
0.61	0.430	0.950	0.430	0.050	0.570	0.380	0.005
0.88	0.016	1.000	0.016	0.001	0.984	0.015	0.012

### Variable Importance

To estimate variable importance, we used SHAP (SHapley Additive exPlanations), a relatively new method in machine learning for interpreting model predictions [32]. SHAP is an approach grounded in principles of cooperative game theory that provides both global and local estimates of how much each feature in the model contributes to obtaining the model output. It considers all possible feature combinations and calculates the difference between the prediction and the average prediction across all combinations. For a given prediction, SHAP assigns a value to each feature, indicating how much that feature contributed to the deviation of the prediction from the baseline. These local values can be positive or negative, depending on whether they increase or decrease the prediction. A mean absolute SHAP value is then calculated for each feature by aggregating the local values across all predictions to provide a global estimate of the feature importance.

As a secondary analysis, we estimated feature importance using mean decrease in accuracy (MDA). MDA, also known as “permutation importance,” is one of the oldest feature importance methods [21]. It provides an estimate of the contribution of each variable to the accuracy of the model by permuting and averaging the decrease in accuracy over all trees in the forest with the permuted feature values as compared to the initial accuracy of the model with all features. In addition to calculating overall MDA, we estimate MDA for only the minority class observations. This allows us to better understand the model’s accuracy in predicting crime gun recovery specifically.

## Results

For the model predicting any crime gun recovery within a year, the test set AUC is 0.85. Table 1 presents sensitivity, specificity, and other performance metrics for a range of thresholds. With a default threshold of .50, sensitivity is .63 and specificity is .88. Figure 1 provides a graphical representation of the predictions for crime guns and non-crime guns, illustrating the trade-offs between false positives and false negatives as the threshold moves along the *x*-axis.

Results are similar for the prediction of crime gun recovery in a violent crime within a year. The test set AUC is 0.85; with a default threshold of 0.50, sensitivity is 0.66, and specificity is 0.88 (Table 2).

**Fig. 2:**
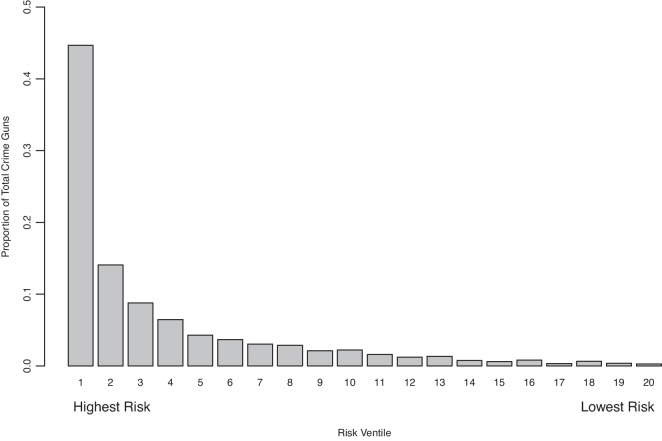
Crime gun recovery by ventile of predicted risk

**Fig. 3:**
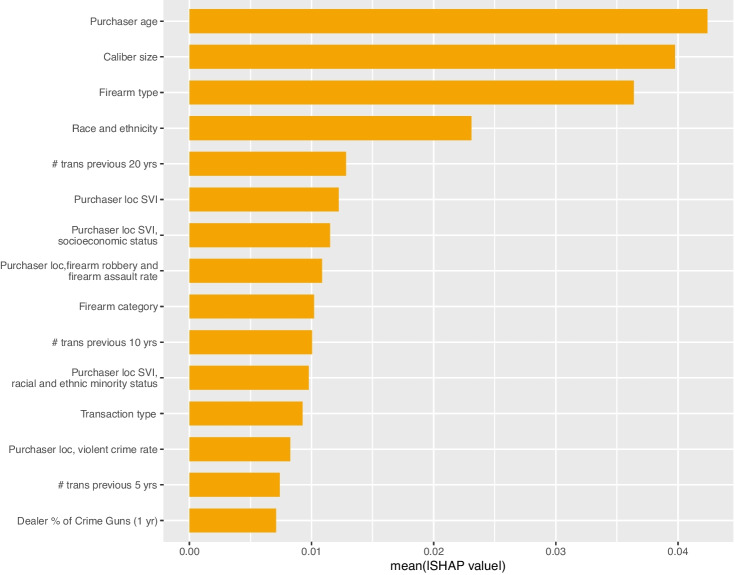
Global SHAP values: crime gun recovery within 1 year

Figure 2 shows the probabilities from the any crime gun model, ranked from highest to lowest risk and grouped into equal size ventiles with the observed percentage of crime guns on the *y*-axis. Close to half (45%) of all transactions that become a crime gun within a year are in the top 5% of predicted risk. Results are similar for the violent crime gun model: 43% of the riskiest 5% of transactions were recovered in a violent crime within a year.

In the model predicting any recovery, we do particularly well at identifying extremely high-risk transactions. For example, among the small number of transactions with a RF score of 0.98 and above, more than three-quarters of these transactions (35 out of 47 in the test data) were recovered in crime within a year.

**Fig. 4:**
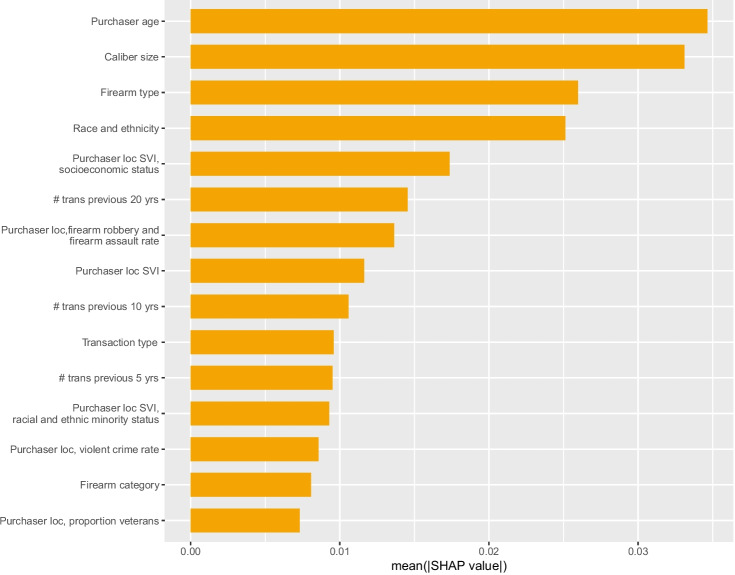
Global SHAP values: violent crime gun recovery within 1 year

Results from the secondary analyses are comparable to our primary models. The test set AUC for the model predicting crime gun recovery excluding race/ethnicity is .84; the AUC is slightly lower for the model including only handguns (.82) and the model including a reduced set of predictors (.83). Additional evaluation metrics are shown in the Supplement (Tables A3, A5, A6, A4).

### Variable Importance

As shown in Figs. 3 and 4, the top four global SHAP values for both the any crime gun recovery and the violent crime gun recovery models are the same: purchaser age is the most important feature followed by caliber size, firearm type, and purchaser race/ethnicity. Table 3 presents the mean values of select input features that are significantly different between crime guns and non-crime guns. The full set of features are presented in the Supplement (Table A1), as are the purchaser, firearm, transaction, and community characteristics for firearms recovered in a violent crime within a year as compared to all other firearms (Table A2). The Supplement also presents two examples of local SHAP importance in the Supplement (Figs. A5 and A6)

The average age for short time-to-crime gun purchasers is 34 (median 30) as compared to an average age of 44 (median 42) among purchasers whose firearms were not recovered within a year (Table 3). Overall, 84% of short time-to-crime guns are pistols as compared to 61% among transactions that were not recovered within a year. Short time-to-crime guns are more likely to be medium and large caliber (44% were large caliber and 34% medium caliber as compared to 31% and 23%, respectively, among non-crime guns). Firearms that were not recovered in crime within a year are more likely to be center-fire rifles (6% vs 15%). Finally, purchasers of short time-to-crime guns purchasers are more likely to be Black (19% vs 4%) or Hispanic (30% vs 17%) and less likely to be white (39% vs 64%).

Following these top four features, the remaining top SHAP values are similar in magnitude. For the model predicting any recovery within a year, the next most important features are the number of transactions that the purchaser made in the past 20 years followed by the Social Vulnerability Index (SVI) associated with the purchaser’s address. For violent crime gun recovery, both SVI and the number of transactions in the past 20 years are important, followed by the city firearm robbery and assault rate associated with the purchasers’ address. Again, we see that these features are significantly different among gun purchasers whose firearm was recovered within a year and those whose firearm was not recovered (Table 3). All measures of SVI are substantially higher for those whose firearm purchase was recovered in a crime within a year (e.g., a socioeconomic SVI of 60 vs 45). The crime rates are also significantly higher: a firearm robbery and assault rate of 136 per 10,000 vs a rate of 90 per 100,000).

**Table 3:** Select descriptive statistics (crime gun vs non-crime gun)

		Non crime gun	Crime gun
**Select firearm characteristics**			
*Firearm type*			
	Pistol	61.5%	84.4%
	Rifle	27.9%	9.7%
*Firearm category*			
	Revolver	9.7%	9.6%
	Semi-automatic	71.0%	82.6%
	Bolt action	6.8%	1.8%
*Caliber size*			
	Small	6.5%	6.0%
	Medium	31.2%	44.3%
	Large	23.0%	34.0%
	Centerfire rifle	15.4%	6.4%
	Rimfire rifle	5.6%	2.9%
	Rifle, frame only	6.2%	0.4%
	Shotgun, not 410	10.3%	5.8%
*Inexpensive firearm (proxy)*			
	Cheap manufacturer	1.5%	5.8%
**Select dealer characteristics**			
*% of sales that were crime guns (past year)*			
	None	43.7%	30.5%
	Some	42.0%	41.1%
	Top quartile	14.3%	28.4%
**Select purchaser characteristics**			
*Race/ethnicity*			
	Black	3.6%	19.2%
	Hispanic	17.3%	30.2%
	Other or unknown	15.0%	11.9%
	White	64.2%	38.7%
*Sex*			
	Female	9.0%	16.0%
	Male	89.4%	82.9%
	Unknown	1.8%	1.1%
*Age*			
		43.8 (14.4)	34.4 (12.9)
*Criminal history (arrest in the past 30 years)*			
	Alcohol-related	7.1%	14.0%
	Drug-related	2.8%	10.2%
	Major property crime	1.8%	7.0%
	Violent crime	4.6%	14.0%
	Firearm-related	1.6%	4.9%
*# Transactions previous 20 yrs*		4.6 (20.5)	2.7 (14.5)
**Select community characteristics**			
*Social Vulnerability Index (purchaser census tract)*			
	Overall	43.8 (26.1)	59.0 (27.4)
	Socioeconomic status	44.8 (26.0)	59.5 (27.1)
	Racial and ethnic minority status	41.0 (25.9)	55.9 (27.1)
*Community crime rates (purchaser city)*			
	Firearm robbery + assault rate	90.4 (317.6)	136.0 (317.6)
	Violent crime rate	482.4 (1434.3)	613.6 (1405.3)

Our estimates of variable importance using MDA are generally similar to those using SHAP, particularly MDA calculated specifically for the minority class (crime guns). The exception is purchaser criminal history variables, which are among the most important features calculated using MDA (Supplement Figs. A1 A2, A3, A4). Descriptively, we observe significant differences in purchaser criminal history between those whose guns were recovered within a year and those that were not. For any crime gun recovery, the fraction of purchasers with a prior arrest for a firearm-related crime, violent crime, or drug crime is roughly three times that of purchasers whose firearm was not recovered within a year (4.9% vs 1.6%, 14.0% vs 4.6%, and 10.2% vs 2.8%, respectively); alcohol-related arrest is double among those whose firearms were recovered within a year (14.0% vs 7.1%).

Criminal history variables also appear as important, calculated via SHAP, in the secondary analysis with a reduced predictor set: any prior violent crime arrest and any alcohol-related arrest both appear in the top 15 SHAP features (Fig. A7). Purchaser sex is also among the top SHAP values in this reduced predictor set model. Otherwise, the most important features in this secondary analysis are comparable to those in the primary models (Supplement Figs. A8 and A9).

## Discussion

Overall, the models that we built show good discrimination and are able to relatively accurately identify firearms that are at the highest risk for being diverted from the legal market for criminal use soon after purchase. The model predicting any crime gun recovery within a year performs particularly well at identifying a small number of extremely risky transactions.

In addition to developing risk prediction models, we identified important predictors of short time-to-crime gun recovery and short time to recovery in a violent crime. This machine learning variable importance estimation is an alternative to the standard multivariate parametric modeling approach that has traditionally been used to identify crime gun risk factors. A machine learning approach can better assess combinations of features that are most predictive of recovery. Nonetheless, the features that we identified as most important (e.g., caliber size, firearm type, purchaser age, and race/ethnicity) were largely consistent with those documented in the previous crime gun research. For example, research on crime gun recoveries in Baltimore, MD, in the 1990s, found the hazard for medium caliber handguns was 56% higher than that for small handguns, and handguns were four times more likely to be recovered if the purchaser was Black and significantly more likely to be recovered if the purchaser was young [8]. In a more recent multivariate survival analysis of crime guns in California over the last decade, we similarly found these variables were positively associated with crime gun recovery [13].

Importantly, though race/ethnicity appears as an important predictor, we achieve comparable performance when we generate a RF model excluding race/ethnicity. This likely reflects the fact that many features in the algorithm are highly correlated. This also underscores the fact that the variable importance measures merely point to features that are *predictive* but do not provide information on causal relationships. It is important to note that though race is predictive of crime gun recovery, we cannot disambiguate the extent to which this reflects other important correlated features, racial disparities in surveillance practices and police behavior, and/or differential unlawful behavior.

To our knowledge, the present study, and our related survival analysis [13], are the first crime gun studies to include purchaser criminal history. We find these predictors are important when we estimate variable importance via MDA and are important SHAP features in the reduced predictor set model with indicators for any arrest within 30 years. This finding of importance is consistent with previous research showing a strong association between legal firearm purchaser criminal history and the likelihood that they will perpetrate a subsequent offense. For example, individuals with a history of DUI conviction have been shown to be at substantially higher risk of subsequent arrest for violent crimes [33]. In the crime gun survival analysis, we found a purchaser’s previous criminal history increased the hazard of a handgun becoming a crime gun by a factor of approximately two [13]. Despite the fact that research suggests that most weapons used in crime are not directly acquired by the perpetrator from a licensed dealer, the characteristics of the last recorded purchaser that are predictive of crime gun recovery are consistent with well-documented risk factors for criminal participation [10, 34].

The algorithms that we developed in this study are proof of concept, nonetheless, risk prediction such as this could potentially aid trafficking and violence prevention efforts. For example, a risk prediction tool could flag high-risk firearm sales and allow for intervention at the time of purchase or during the 10-day waiting period. For instance, ATF, in partnership with the firearm industry’s trade association (The National Shooting Sports Foundation), has a program, “Don’t Lie for the Other Guy,” that is designed to assist firearm retailers in the detection and possible deterrence of straw purchases [35]. This includes a public awareness campaign warning about the seriousness of the crime of purchasing a firearm for someone who cannot legally do so, and efforts to help firearms retailers better identify potential straw purchasers. A risk prediction tool might serve as an empirically driven supplement to the current list of “red flags” that retailers are meant to look out for. Another possibility is that a higher risk score could prompt a letter during the mandatory waiting period between purchase and pick up (in states that impose waiting periods), reminding purchasers of the laws prohibiting straw purchasing. A previous randomized control trial in California showed that a letter sent during California’s 10-day waiting period to individuals thought to be potential straw purchasers stating sanctions for violations of legal obligations led to a higher rate at which guns were reported stolen among those who received the letter, although it did not change the rate at which the firearms were picked up [36].

### Limitations

There are a number of limitations to note. First, as is the case with any research relying on trace data, the analyses are focused on *legal* firearm transactions and subsequent recovery by law enforcement. Many firearms that are used in crimes are never recovered by the police. We are necessarily predicting law enforcement recovery in crime as opposed to criminal use more broadly.

Among firearms that are recovered, we do not have information on the full life course of the gun, including any illegal secondary transfers between the last retail sale and recovery. We do have data on firearms that are reported lost or stolen following a sale. Stolen firearms are substantially more likely to be recovered in crime [13]. However, we do not include theft as a predictor because this sort of risk prediction tool would likely be most useful at the point of purchase or during the 10-day waiting period, before a future theft could be known. For this same reason, we retain these observations in our models. Removing transactions reported lost or stolen within a year (0.8% of transactions; 6.9% of firearms recovered in a year) does not change model performance or our variable importance measures.

An additional limitation specific to the CA DOJ data is that the records do not include responses from the ATF to local law enforcement trace requests. Given Tiahrt Amendment prohibitions, we do not have access to ATF trace results and therefore do not have information on out-of-state purchases. This is necessarily a within-state study of transactions and recoveries in California. According to ATF trace data reports over the past decade, between half to almost three-quarters of recovered crime guns in California were first purchased in the state (when a source state was identified) [37]. Though we do not have out-of-state transactions or recoveries, unlike ATF trace data studies, we do have information on *all* legal transactions for a given firearm, whereas trace data only include the *first* recorded purchase.

Our analyses, limited to California, may not generalize. California is a state with particularly stringent gun laws. For example, California has more criminal prohibitions on purchase and possession than most states, such as prohibiting those with a misdemeanor violent crime conviction from acquiring or possessing a firearm [38]. California also limits the number of firearms that can be purchased to one per month. Prior literature has found that multiple purchases within a day are a strong indicator of a crime gun purchase [8].

California is also unique in that it is the only state that currently records, maintains, and makes available for research records of all firearm transactions conducted in the state as well as law enforcement crime gun recovery records. In theory, however, risk prediction models such as we have generated could be developed in other states. Eleven other states have implemented policies that require law enforcement to trace firearms used in crimes (Connecticut, Delaware, Hawaii, Illinois, Maryland, Massachusetts, New Jersey, New York, Ohio, Oregon, and Pennsylvania) [39], though these states do not clearly centralize and maintain these records as California does. Five states besides California require licensed dealers to report all firearm transactions to law enforcement (Connecticut, Hawaii, Massachusetts, Oregon, and Rhode Island), and another six require the reporting of handgun transactions only (Maryland, Michigan, New Jersey, New York, Pennsylvania, Washington) [40].

Real-world model implementation of this sort of risk prediction would require addressing practical considerations. Our aim was to maximize predictive accuracy. However, were an agency such as CA DOJ to implement a risk prediction model, it might be more practical to, for example, exclude community characteristics. We obtained these features by geocoding addresses and linking associated variables from sources including the US Census and the FBI UCR crime reports. On the other hand, the variables related to the transaction, firearm and individual purchaser, were all derived directly from CA DOJ data.

A final important and general limitation to note is that, while our models are informative, they are imperfect. Crime gun recovery within a year is an extremely rare event, and even a high prediction threshold includes many false positives. The risk predictions are useful only in ranking and identifying the highest risk to potentially deploy additional scrutiny.

## Conclusion

Understanding which firearms end up being diverted from the legal market and used in crime shortly after sale can inform efforts to reduce the flow of guns into illicit markets and criminal hands. This is the first study to employ machine learning to identify transactions at high risk of being recovered soon after purchase and the features most predictive of recovery. The results suggest the potential utility of large-scale firearm purchasing and law enforcement recovery data to identify risky sales and the risk factors associated with crime gun recovery.

## Supplementary Information

Below is the link to the electronic supplementary material.

Supplementary file 1 (pdf 176 KB)

## Data Availability

The data used in this study are available from the California Department of Justice. Restrictions apply to the availability and sharing of these data, which were used under license for the current study, and so are not publicly available.
