# Supplementary material for: Predicting Short Time-to-Crime Guns: a Machine Learning Analysis of California Transaction Records (2010–2021)
Source: J Urban Health. 2024 Sep 5;101(5):955–67. doi: 10.1007/s11524-024-00909-0 (PMC11461422; doi:10.1007/s11524-024-00909-0)
Supplement: Supplementary file 1 — (pdf 176 KB) [file 11524_2024_909_MOESM1_ESM.pdf]

Supplement

A Summary Tables for Predictor Variables

Table A1: Descriptive statistics: crime gun (recovered within 1 year) vs non-crime gun

	Non Crime Gun (7,802,417)	Crime Gun (15,945)
**Firearm Characteristics**		
Firearm type, Pistol	61.53%	84.38%
Firearm type Rifle	27.86%	9.74%
Firearm type, Shotgun	10.51%	5.86%
Firearm type, Rifle-shotgun combination	0.09%	0.01%
Firearm type , Other	0.01%	0.00%
Firearm category, Semi-automatic	70.88%	82.63%
Firearm category, Revolver	9.73%	9.60%
Firearm category, Bolt action	6.77%	1.81%
Firearm category, Pump action	6.52%	4.80%
Firearm category, Other	3.83%	0.76%
Firearm category, Single shot	2.26%	0.40%
Caliber size, Handgun, frame only	0.28%	0.01%
Caliber size, Handgun, interchangeable barrel	0.50%	0.01%
Caliber size, Small	6.46%	6.03%
Caliber size, Medium	31.21%	44.33%
Caliber size, Large	23.03%	34.00%
Caliber size, Center-fire rifle	15.44%	6.38%
Caliber size, Rim-fire rifle	5.64%	2.91%
Caliber size, Rifle, frame only	6.15%	0.36%
Caliber size, Rifle, interchangeable barrel	0.56%	0.09%
Caliber size, Rifle-shotgun combination	0.09%	0.01%
Caliber size, Shotgun, frame only	0.04%	0.00%
Caliber size, Shotgun, interchangeable barrel	0.02%	0.00%
Caliber size, Shotgun, 410	0.24%	0.11%
Caliber size, Shotgun, not 410	10.27%	5.76%
Caliber size, Other or Unknown	0.06%	0.01%
Cheap make firearm	1.52%	5.75%
**Transaction Information**		
Transaction type , Curio or relic	6.99%	2.31%
Transaction type, Non-roster peace officer	2.27%	0.43%
Transaction type, Other or unknown	1.49%	0.70%
Transaction type, Private party transfer	14.30%	11.30%
Transaction type, Pawn redemption	1.21%	1.83%
Transaction type, Dealer sale	73.75%	83.44%
Document code, Dealer's record of sale	98.77%	98.17%
Document code, Pawn redemption	1.23%	1.83%
Transaction status, Approved	98.23%	98.91%
Transaction status, Canceled or denied	0.28%	0.40%
Transaction status, Unknown	1.49%	0.70%
Gun show	1.15%	1.42%
Purchase month, January	7.36%	7.07%
Purchase month , February	7.52%	8.82%
Purchase month, March	9.16%	10.57%
Purchase month, April	7.80%	8.67%
Purchase month, May	7.39%	8.55%
Purchase month, June	7.79%	7.64%
Purchase month, July	7.61%	7.81%

Purchase month, August	8.08%	8.18%
Purchase month, September	7.50%	7.30%
Purchase month, October	8.30%	8.05%
Purchase month, November	9.60%	8.71%
Purchase month, December	11.89%	8.63%
ID type, CA driver's license	96.28%	90.68%
ID type, CA ID card	1.00%	7.28%
ID type, DOD ID card	0.73%	0.68%
ID type, Military ID card	0.22%	0.30%
ID type, Unknown	1.77%	1.07%
Police/military flag	14.17%	10.47%
**Purchaser Information**		
Purchaser age	43.79 (14.37)	34.40 (12.86)
White	64.21%	38.66%
Hispanic	17.26%	30.19%
Black	3.56%	19.22%
Other or unknown race	14.97%	11.93%
Male	89.35%	82.92%
Female	8.88%	16.02%
Unknown sex	1.77%	1.07%
# trans previous 180 days	0.64 (3.49)	0.69 (5.03)
# trans previous 1 yr	1.06 (5.18)	1.16 (7.66)
# trans previous 5 yrs	2.90 (13.55)	2.18 (12.94)
# trans previous 10 yrs	3.79 (17.38)	2.48 (13.77)
# trans previous 20 yrs	4.57 (20.48)	2.74 (14.51)
Purchaser denial within past 90 days	0.63%	0.85%
Purchaser denial within past 180 days	0.92%	1.21%
Purchaser denial within past 1 year	1.40%	1.76%
Purchaser denial within past 5 years	3.27%	3.15%
Foreign born	16.23%	13.46%
Not a citizen	2.03%	3.13%
**Purchaser Criminal History**		
Drug-related arrest in past 1 year	0.05%	0.70%
Drug-related arrest in past 5 years	0.40%	4.08%
Drug-related arrest in past 30 years	2.80%	10.23%
Major property crime arrest in past 1 year	0.01%	0.14%
Major property crime arrest in past 5 years	0.11%	1.17%
Major property crime arrest in past 30 years	1.78%	6.98%
Violent crime arrest in past 1 year	0.09%	1.07%
Violent crime arrest in past 5 years	0.59%	4.30%
Violent crime arrest in past 30 years	4.57%	13.95%
Alcohol-related arrest in past 1 year	0.25%	1.39%
Alcohol-related arrest in past 5 years	1.86%	6.50%
Alcohol-related arrest in past 30 years	7.08%	13.99%
Firearm violence arrest in past 1 year	0.01%	0.13%
Firearm violence arrest in past 5 years	0.04%	0.28%
Firearm violence arrest in past 30 years	0.34%	0.80%
Firearm-related arrest in past 1 year	0.05%	0.92%
Firearm-related arrest in past 5 years	0.24%	2.15%
Firearm-related arrest in past 30 years	1.57%	4.93%
Major violent crime arrest in past 1 year	0.04%	0.50%
Major violent crime arrest in past 5 years	0.26%	1.89%
Major violent crime arrest in past 30 years	2.36%	7.24%
**Purchaser Address Census Tract Information**		
Most urban	82.65%	91.22%
Most rural	2.13%	0.73%
SVI	43.83 (26.13)	58.75 (27.35)
SVI, socioeconomic status	44.78 (26.00)	59.51 (27.08)
SVI, housing status	52.46 (27.99)	58.06 (28.42)

SVI, racial and ethnic minority status	40.95 (25.94)	55.93 (27.08)
SVI, housing type and transportation	43.27 (27.54)	53.78 (27.87)
Proportion foreign born	0.21 (0.13)	0.26 (0.13)
Proportion veterans	0.08 (0.04)	0.06 (0.04)
Property crime rate	3810.37 (20959.12)	4038.36 (19835.92)
Firearm robbery and firearm assault rate	90.41 (317.55)	135.65 (317.59)
Violent crime rate	482.44 (1434.26)	613.56 (1405.23)
Ratio of M aged 15-20 to M aged 40-44	1.25 (2.63)	1.18 (0.48)
Ratio M-F aged 15-30	1.10 (1.04)	1.10 (1.74)
**Dealer Information**		
% Pawn, None	95.00%	93.56%
% Pawn, Some	2.61%	3.58%
% Pawn, Top	0.90%	2.16%
% Pawn, Unknown	1.49%	0.70%
% Pawn Redemption, None	55.61%	48.28%
% Pawn Redemption, Some	32.13%	37.65%
% Pawn Redemption,Top	10.78%	13.38%
% Pawn Redemption, Unknown	1.49%	0.70%
% Crime Guns (1 yr), None	43.68%	30.50%
% Crime Guns (1 yr), Some	41.96%	41.10%
% Crime Guns (1 yr), Top	14.26%	28.40%
% Crime Guns (1 yr), Unknown	0.10%	0.00%
Denial rate	0.03 (0.09)	0.03 (0.07)
Dealer sales volume	1141.72 (1141.91)	1394.09 (1255.76)
**Dealer Address Census Tract Information**		
Most urban	75.12%	79.45%
Most rural	0.93%	0.24%
SVI	50.48 (25.10)	55.32 (25.05)
SVI, socioeconomic status	49.09 (24.82)	53.72 (24.46)
SVI, housing status	53.44 (30.13)	54.95 (30.26)
SVI, racial and ethnic minority status	43.98 (23.45)	49.91 (23.14)
SVI, housing type and transportation	54.66 (27.60)	57.68 (27.57)
Proportion foreign born	0.23 (0.12)	0.25 (0.12)
Proportion veterans	0.07 (0.04)	0.06 (0.03)
Property crime rate	4579.26 (27631.72)	5037.88 (30799.17)
Firearm robbery and firearm assault rate	94.56 (461.26)	123.17 (501.78)
Violent crime rate	495.06 (1862.79)	579.33 (2113.62)
Ratio of M aged 15-20 to M aged 40-44	1.12 (0.45)	1.07 (0.42)
Ratio M-F aged 15-30	1.08 (0.20)	1.08 (0.25)
**Other Variables**		
Distance from purchaser to dealer	31.12 (71.72)	26.74 (70.35)
Statewide monthly average purchases	37555.49 (7407.56)	36763.98 (7914.36)

Table A2: Descriptive statistics: violent crime gun vs non crime gun

	Non Crime Gun (7,816,230)	Violent Crime Gun (2,132)
**Firearm Characteristics**		
Firearm type, Pistol	61.57%	82.83%
Firearm type, Rifle	27.83%	10.88%
Firearm type, Shotgun	10.50%	6.29%
Firearm type, Rifle-shotgun combination	0.09%	0.00%
Firearm type, Other	0.01%	0.00%
Firearm category, Semi-automatic	70.90%	82.46%
Firearm category, Revolver	9.73%	9.47%
Firearm category, Bolt action	6.76%	1.74%
Firearm category, Pump action	6.52%	5.16%
Firearm category, Other	3.83%	0.84%
Firearm category, Single shot	2.26%	0.33%

Caliber size, Handgun, frame only	0.28%	0.00%
Caliber size, Handgun, interchangeable barrel	0.50%	0.00%
Caliber size, Small	6.46%	6.71%
Caliber size, Medium	31.23%	44.23%
Caliber size, Large	23.05%	31.89%
Caliber size, Center-fire rifle	15.43%	6.85%
Caliber size, Rim-fire rifle	5.64%	3.94%
Caliber size, Rifle, frame only	6.14%	0.05%
Caliber size, Rifle, interchangeable barrel	0.56%	0.05%
Caliber size, Rifle-shotgun combination	0.09%	0.00%
Caliber size, Shotgun, frame only	0.04%	0.00%
Caliber size, Shotgun, interchangeable barrel	0.02%	0.00%
Caliber size, Shotgun, 410	0.24%	0.05%
Caliber size, Shotgun, not 410	10.26%	6.24%
Caliber size, Other or unknown	0.06%	0.00%
Cheap make firearm	1.53%	6.80%
**Transaction Information**		
Transaction type, Curio or relic	6.98%	1.74%
Transaction type, Non-roster peace officer	2.27%	0.42%
Transaction type, Other or unknown	1.49%	1.17%
Transaction type, Private party transfer	14.30%	9.10%
Transaction type, Pawn redemtion	1.21%	1.69%
Transaction type, Dealer sale	73.76%	85.88%
Document code, Dealer's record of sale	98.77%	98.31%
Document code, Pawn redemption	1.23%	1.69%
Transaction status, Approved	98.24%	98.64%
Transaction status, Canceled or denied	0.28%	0.19%
Transaction status, Unknown	1.49%	1.17%
Purchase month, January	7.36%	6.85%
Purchase month, February	7.52%	7.88%
Purchase month, March	9.17%	11.16%
Purchase month, April	7.80%	9.05%
Purchase month, May	7.39%	7.97%
Purchase month, June	7.79%	7.32%
Purchase month, July	7.61%	7.69%
Purchase month, August	8.08%	8.82%
Purchase month, September	7.49%	8.11%
Purchase month, October	8.30%	7.79%
Purchase month, November	9.60%	9.19%
Purchase month, December	11.88%	8.16%
ID type, CA driver's license	96.27%	91.65%
ID type, CA ID card	1.01%	6.33%
ID type, DOD ID card	0.73%	0.42%
ID type, Military ID card	0.22%	0.23%
ID type, Unknown	1.76%	1.36%
Police/military flag	14.16%	9.94%
Gun show	1.15%	1.50%
**Purchaser Information**		
Purchaser age	43.78 (14.37)	34.73 (12.85)
White	64.17%	36.68%
Hispanic	17.28%	30.91%
Black	3.59%	19.32%
Other or unknown race/ethnicity	14.96%	13.09%
Male	89.34%	83.54%
Female	8.89%	15.10%
Unknown sex	1.76%	1.36%
# trans previous 180 days	0.64 (3.49)	0.24 (0.80)
# trans previous 1 yr	1.06 (5.19)	0.39 (1.49)
# trans previous 5 yrs	2.90 (13.55)	0.90 (4.58)

# trans previous 10 yrs	3.79 (17.37)	1.15 (5.96)
# trans previous 20 yrs	4.56 (20.47)	1.34 (7.01)
Purchaser denial within past 90 days	0.63%	0.70%
Purchaser denial within past 180 days	0.93%	1.03%
Purchaser denial within past 1 year	1.40%	1.27%
Purchaser denial within past 5 years	3.27%	2.53%
Foreign born	16.23%	15.95%
Not a citizen	2.03%	3.94%
**Purchaser Criminal History**		
Drug-related arrest in past 1 year	0.05%	0.28%
Drug-related arrest in past 5 years	0.41%	3.47%
Drug-related arrest in past 30 years	2.82%	9.57%
Major property crime arrest in past 1 year	0.01%	0.05%
Major property crime arrest in past 5 years	0.11%	0.89%
Major property crime arrest in past 30 years	1.79%	6.29%
Violent crime arrest in past 1 year	0.09%	1.36%
Violent crime arrest in past 5 years	0.59%	4.88%
Violent crime arrest in past 30 years	4.59%	15.62%
Alcohol-related arrest in past 1 year	0.25%	1.13%
Alcohol-related arrest in past 5 years	1.87%	6.14%
Alcohol-related arrest in past 30 years	7.09%	13.79%
Firearm violence arrest in past 1 year	0.01%	0.14%
Firearm violence arrest in past 5 years	0.04%	0.33%
Firearm violence arrest in past 30 years	0.34%	0.75%
Firearm-related arrest in past 1 year	0.05%	0.94%
Firearm-related arrest in past 5 years	0.25%	1.97%
Firearm-related arrest in past 30 years	1.57%	4.64%
Major violent crime arrest in past 1 year	0.04%	0.66%
Major violent crime arrest in past 5 years	0.26%	2.02%
Major violent crime arrest in past 30 years	2.37%	7.69%
**Purchaser Address Census Tract Information**		
Most urban	82.66%	91.60%
Most rural	2.13%	0.56%
SVI	43.85 (26.14)	59.67 (27.33)
SVI, socioeconomic status	44.80 (26.01)	60.30 (26.83)
SVI, housing status	52.47 (27.99)	58.91 (28.58)
SVI, racial and ethnic minority status	40.98 (25.95)	56.23 (26.94)
SVI, housing type and transportation	43.29 (27.55)	54.61 (28.34)
Proportion foreign born	0.21 (0.13)	0.26 (0.13)
Proportion veterans	0.08 (0.04)	0.06 (0.04)
Property crime rate	3810.82 (20956.65)	4009.86 (21153.95)
Firearm robbery and firearm assault rate	90.50 (317.56)	133.08 (319.60)
Violent crime rate	482.69 (1434.22)	615.04 (1387.25)
Ratio of M aged 15-20 to M aged 40-44	1.25 (2.63)	1.18 (0.42)
Ratio M-F aged 15-30	1.10 (1.04)	1.07 (0.14)
**Dealer Information**		
% of Pawn, None	95.00%	93.20%
% of Pawn, Some	2.61%	3.56%
% of Pawn, Top	0.91%	2.06%
% of Pawn, Unknown	1.49%	1.17%
% of Pawn Redemption, None	55.60%	48.50%
% of Pawn Redemption, Some	32.14%	37.99%
% of Pawn Redemption, Top	10.78%	12.34%
% of Pawn Redemption, Unknown	1.49%	1.17%
% of Crime Guns (1 yr), None	43.66%	31.29%
% of Crime Guns (1 yr), Some	41.96%	42.07%
% of Crime Guns (1 yr), Top	14.28%	26.64%
% of Crime Guns (1 yr),Unknown	0.10%	0.00%
Denial rate	0.03 (0.09)	0.03 (0.07)

Sales volume	1142.18 (1142.17)	1406.78 (1268.04)
**Dealer Address Census Tract Information**		
Most urban	75.13%	77.49%
Most rural	0.93%	0.28%
SVI	50.48 (25.10)	55.24 (25.92)
SVI, socioeconomic status	49.10 (24.82)	53.87 (25.31)
SVI, housing status	53.44 (30.13)	55.16 (30.02)
SVI, racial and ethnic minority status	43.99 (23.45)	50.21 (23.21)
SVI, housing type and transportation	54.67 (27.60)	56.94 (28.01)
Proportion foreign born	0.23 (0.12)	0.25 (0.12)
Proportion veterans	0.07 (0.04)	0.06 (0.04)
Property crime rate	4580.28 (27639.17)	4400.89 (26314.91)
Firearm robbery and firearm assault rate	94.61 (461.36)	112.45 (418.10)
Violent crime rate	495.22 (1863.29)	552.48 (2091.65)
Ratio of M aged 15-20 to M aged 40-44	1.12 (0.45)	1.10 (0.43)
Ratio M-F aged 15-30	1.08 (0.20)	1.08 (0.29)
**Other Variables**		
Distance from purchaser to dealer	31.11 (71.72)	26.06 (70.10)
Statewide monthly average purchases	37554.12 (7408.54)	36660.60 (7963.18)

B Model Performance for Additional Models

Table A3: Predicting Any Crime Gun Recovery within 1 Year (Handgun Only)

threshold	sensitivity	specificity	PPV	NPV	Youden	TPR	FPR	FNR	F-score
0.39	0.768	0.745	0.008	0.999	0.513	0.768	0.255	0.232	0.017
0.50	0.605	0.873	0.013	0.999	0.478	0.605	0.127	0.395	0.026
0.63	0.399	0.951	0.022	0.998	0.350	0.399	0.049	0.601	0.042
0.83	0.097	0.996	0.066	0.998	0.094	0.097	0.004	0.903	0.079

Table A4: Predicting Any Crime Gun Recovery within 1 Year (Reduced Set of Predictors)

threshold	sensitivity	specificity	PPV	NPV	Youden	TPR	FPR	FNR	F-score
0.38	0.739	0.751	0.006	0.999	0.490	0.739	0.249	0.261	0.012
0.50	0.579	0.872	0.009	0.999	0.452	0.579	0.128	0.421	0.018
0.64	0.402	0.950	0.017	0.999	0.353	0.402	0.050	0.598	0.032
0.86	0.098	0.997	0.055	0.998	0.094	0.098	0.004	0.902	0.070

Table A5: Predicting Any Crime Gun Recovery within 1 Year (Excluding Race/Ethnicity)

threshold	sensitivity	specificity	PPV	NPV	Youden	TPR	FPR	FNR	F-score
0.44	0.717	0.811	0.008	0.999	0.528	0.717	0.190	0.283	0.015
0.50	0.641	0.867	0.010	0.999	0.508	0.641	0.133	0.359	0.019
0.64	0.425	0.951	0.017	0.999	0.375	0.425	0.050	0.575	0.033
0.84	0.098	0.996	0.045	0.998	0.094	0.098	0.004	0.902	0.062

Table A6: Predicting Violent Crime Gun Recovery within 1 Year (Excluding Race/Ethnicity)

thr	sensitivity	specificity	PPV	NPV	Youden	TPR	FPR	FNR	F-score
0.42	0.7684	0.7811	0.001	0.9999	0.5495	0.7684	0.2189	0.2316	0.0019
0.50	0.6275	0.8732	0.0013	0.9999	0.5007	0.6275	0.1268	0.37	0.0027
0.61	0.4006	0.9501	0.0022	0.9998	0.3507	0.4006	0.0499	0.599	0.0043
0.85	0.0219	0.9989	0.0054	0.9997	0.0208	0.0219	0.0011	0.978	0.0086

C Variable Importance: Mean Decrease in Accuracy

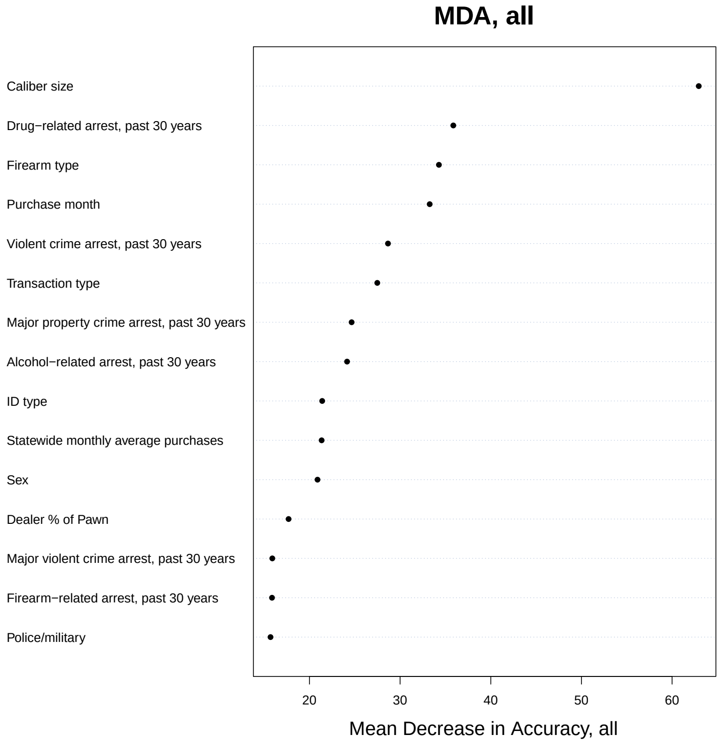

Figure A1: Any Crime Gun Recovery

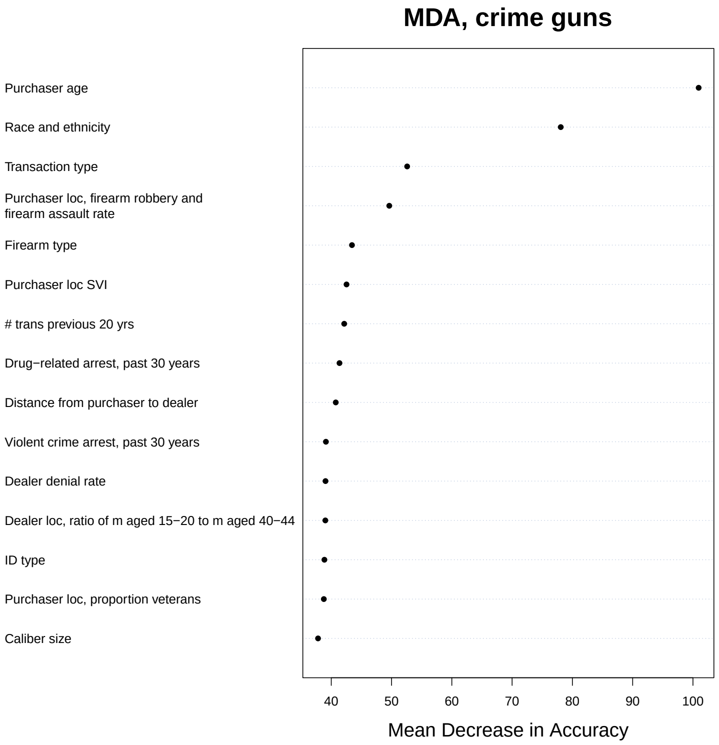

Figure A2: Any Crime Gun Recovery (Minority Class)

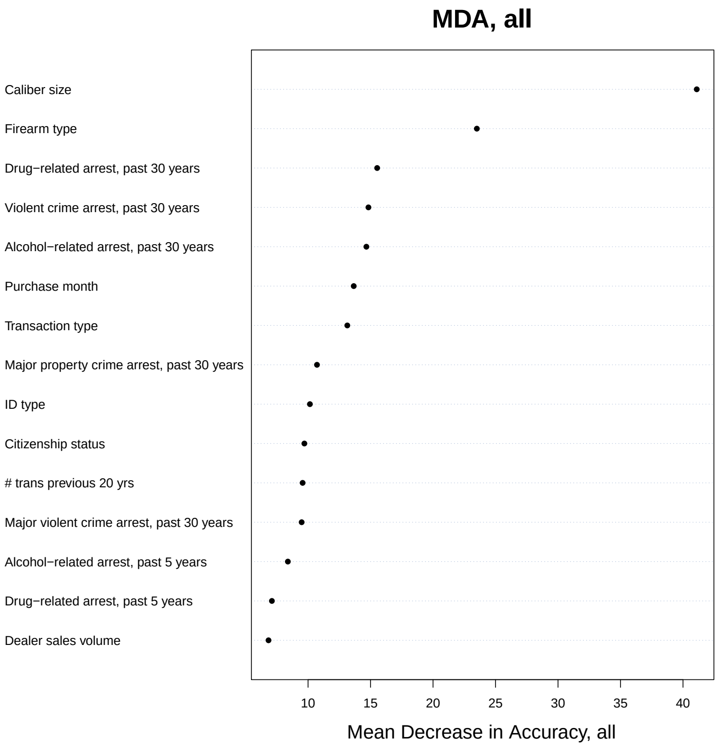

Figure A3: Violent Crime Gun Recovery

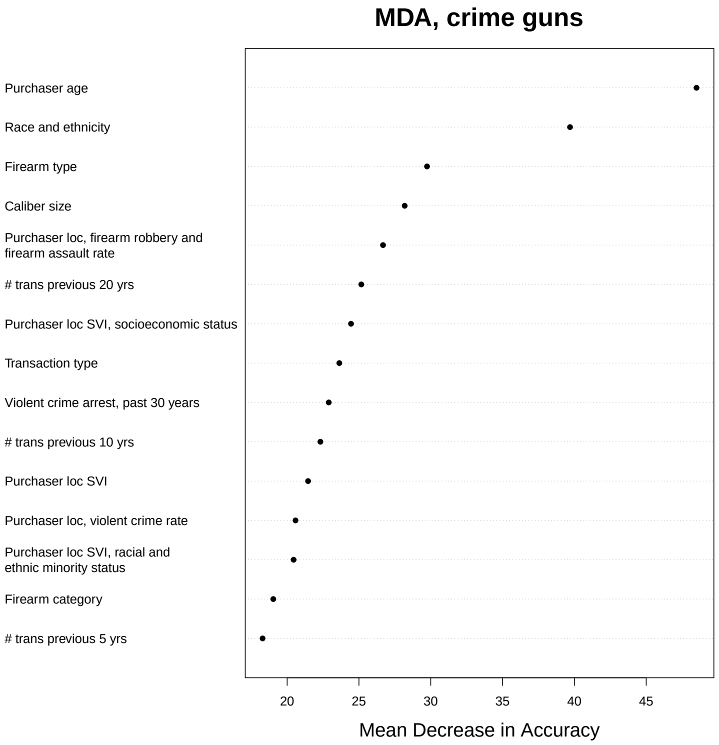

Figure A4: Violent Crime Gun Recovery (Minority Class)

D Variable Importance: Local SHAP Examples

Local SHAP importance is illustrated below with two example waterfall plots for an observation given among the highest RF scores and an observation given one of the lowest. These show the top ten features for the individual observation and its corresponding prediction to help to illuminate the model's decision-making process for that particular instance. A combination of 72 features together account for a little over half of the high risk prediction. Consistent with the global importance measure, we then see several community SVI features as well as community crime rates increase the RF score; the dealer's past year rate of pawn redemptions and rate of denials both increase the score as well. For the lowest risk observations, the "caliber" being a rim-fire rifle reduces the score, as does older purchaser age and the firearm being a revolver.

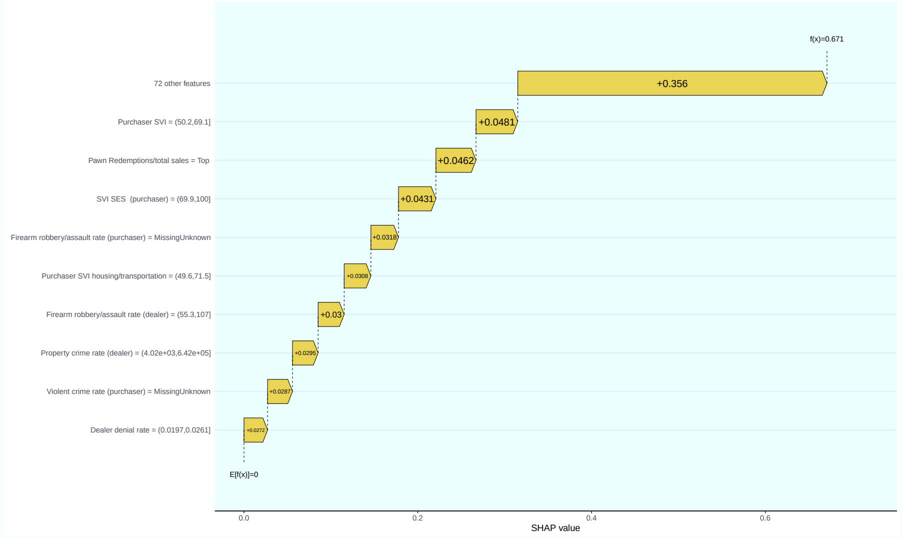

Figure A5: Local SHAP Values: Example High Risk Observation

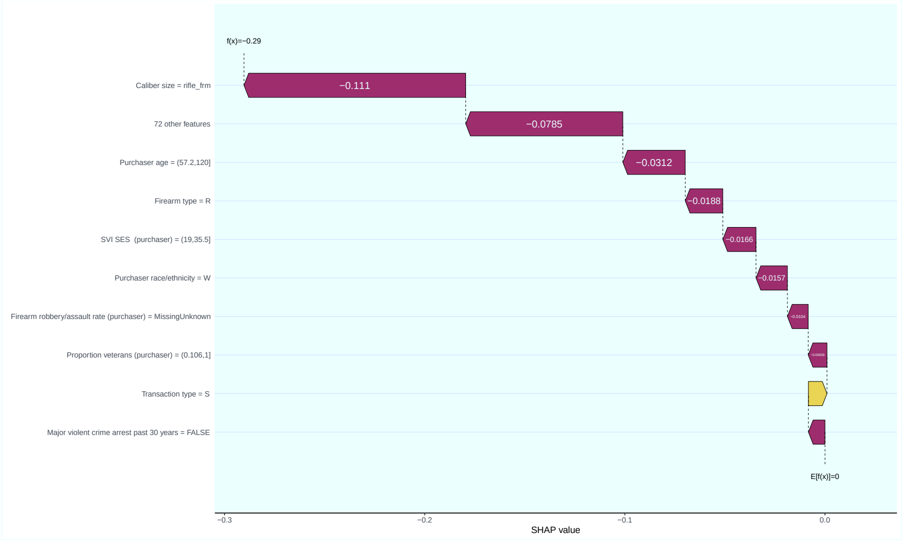

Figure A6: Local SHAP Values: Example Low Risk Observation

Variable Importance: Reduced Set of Predictors (50)

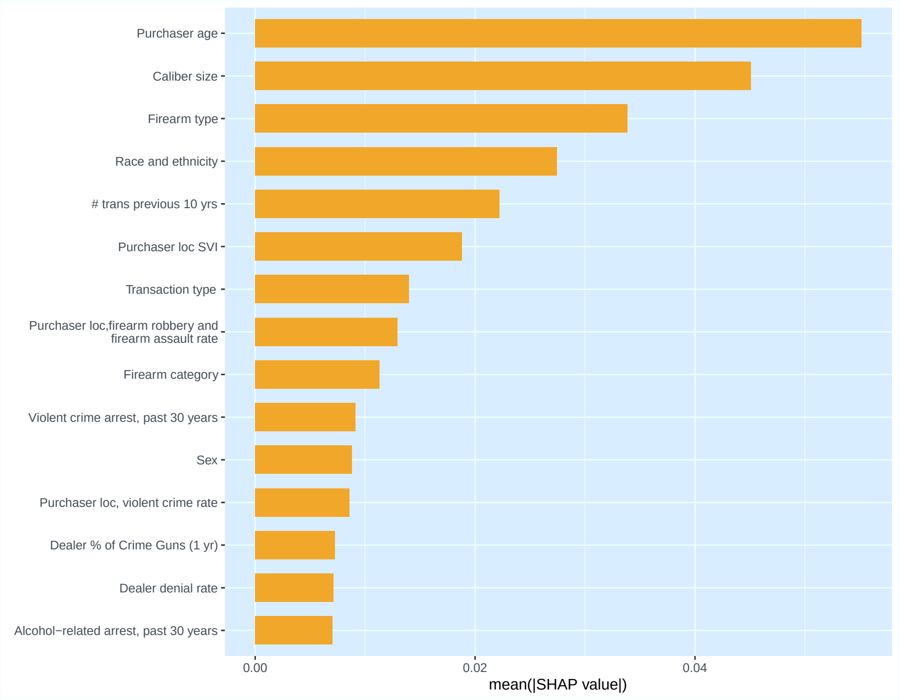

Figure A7: SHAP Values: Any Crime Gun Recovery (Reduced Feature Set)

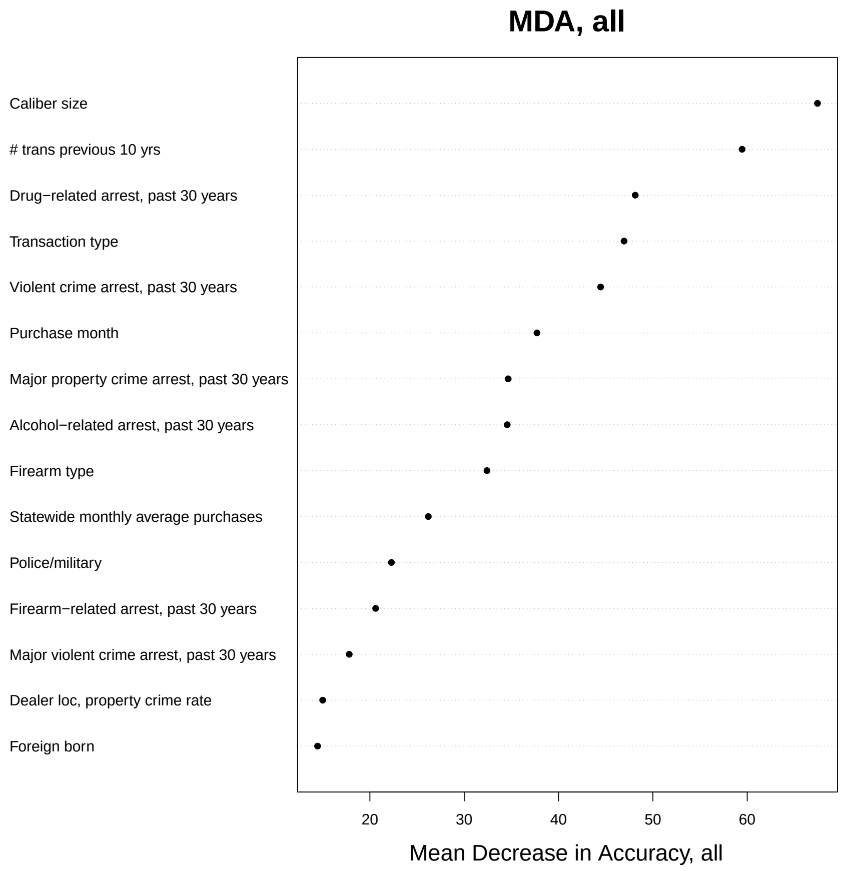

Figure A8: Any Crime Gun Recovery (Reduced Feature Set)

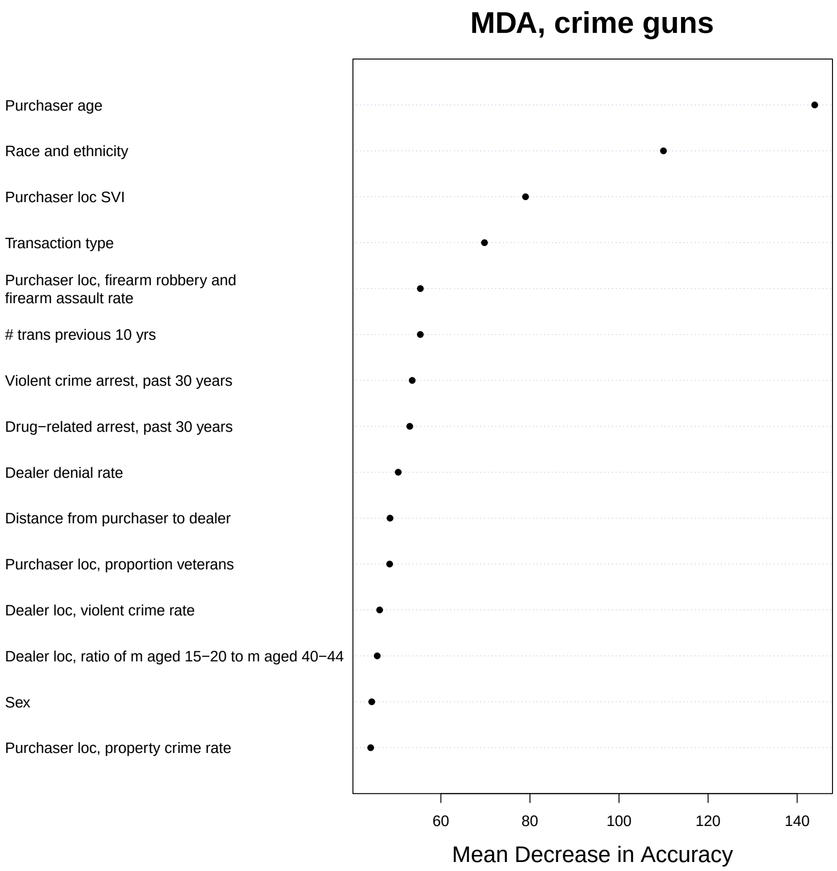

Figure A9: Any Crime Gun Recovery, Minority Class (Reduced Feature Set)
